# The evaluation of distorted body image in anorexia nervosa

**DOI:** 10.1192/j.eurpsy.2022.1491

**Published:** 2022-09-01

**Authors:** N. Ciwoniuk, M. Wayda-Zalewska, K. Kucharska

**Affiliations:** Institute of Psychology; Cardinal Stefan Wyszynski University, Centre Of Psychosomatics And Health Psychology, Warsaw, Poland

**Keywords:** anorexia nervosa, body image

## Abstract

**Introduction:**

A distorted body image appears to be a significant factor predisposing an individual to developing anorexia nervosa and its maintaining. Anorexia nervosa presents with the highest mortality rate among all mental disorders.

**Objectives:**

The aim of the research work was to assess the distorted body image in women diagnosed with anorexia nervosa, as well as to analyse the impact of the severity of the symptomatology of eating disorders, level of depression and anxiety on the distorted body image.

**Methods:**

A total of 105 people participated in the study. The clinical group consisted of 36 women diagnosed with anorexia nervosa, while the group of healthy women consisted of 69 participants. Patients completed several psychological and clinical measures such as: EAT-26, BSQ-34, BIDQ, BDD-YBOCS, CDRS, CESDR, and STAI.

**Results:**

Between group comparisons were performed using nonparametric the Mann-Whitney U test. Results revealed statistically significantly greater distorted body image perception in anorexia group. Women diagnosed with anorexia nervosa showed significantly higher levels both depression and anxiety compared to the healthy controls. Based on correlation analyses, it was shown that there are statistically significant relationships between the body image variable and eating disorders, depression, and anxiety (state and trait).

**Conclusions:**

To deepen the problem of distorted body image, further research is required on etiopathogenesis and dynamics of body image in relation to body mass index and illness chronicity.

**Disclosure:**

No significant relationships.

